# High‐Performance Nonvolatile Organic Field‐Effect Transistor Memory Based on Organic Semiconductor Heterostructures of Pentacene/P13/Pentacene as Both Charge Transport and Trapping Layers

**DOI:** 10.1002/advs.201700007

**Published:** 2017-06-04

**Authors:** Wen Li, Fengning Guo, Haifeng Ling, Peng Zhang, Mingdong Yi, Laiyuan Wang, Dequn Wu, Linghai Xie, Wei Huang

**Affiliations:** ^1^ Key Laboratory for Organic Electronics and Information Displays and Institute of Advanced Materials (IAM) Jiangsu National Synergetic Innovation Center for Advanced Materials (SICAM) Nanjing University of Posts and Telecommunications 9 Wenyuan Road Nanjing 210023 China; ^2^ Key Laboratory of Flexible Electronics (KLOFE) and Institute of Advanced Materials (IAM) Jiangsu National Synergetic Innovation Center for Advanced Materials (SICAM) Nanjing Tech University (NanjingTech) 30 South Puzhu Road Nanjing 211816 China

**Keywords:** flexibility, multilevel, nonvolatile memory, organic field‐effect transistors, organic heterostructures

## Abstract

Nonvolatile organic field‐effect transistor (OFET) memory devices based on pentacene/*N*,*N*′‐ditridecylperylene‐3,4,9,10‐tetracarboxylic diimide (P13)/pentacene trilayer organic heterostructures have been proposed. The discontinuous n‐type P13 embedded in p‐type pentacene layers can not only provide electrons in the semiconductor layer that facilitates electron trapping process; it also works as charge trapping sites, which is attributed to the quantum well‐like pentacene/P13/pentacene organic heterostructures. The synergistic effects of charge trapping in the discontinuous P13 and the charge‐trapping property of the poly(4‐vinylphenol) (PVP) layer remarkably improve the memory performance. In addition, the trilayer organic heterostructures have also been successfully applied to multilevel and flexible nonvolatile memory devices. The results provide a novel design strategy to achieve high‐performance nonvolatile OFET memory devices and allow potential applications for different combinations of various organic semiconductor materials in OFET memory.

Recent advances in the area of organic electronics are mainly due to the wide range of functionalities available from the use of various organic compounds. This has made the development of essential opto/electronic building blocks possible, such as organic light‐emitting diodes,^1^, ^2^, ^3^ organic field‐effect transistors (OFETs),^4^, ^5^, ^6^ and organic photovoltaic cells,^7^, ^8^ leading to emerging applications of organic materials in the fields of microelectronics as well as optoelectronics. The versatility of organic materials has also led to a rising trend in nonvolatile OFET memory research, aiming at potential applications in integrated circuits,^9^ radio‐frequency identification tags,^10^ and portable electronic devices.^11^, ^12^ The use of OFET memory devices directly benefits from their nondestructive read‐out,^13^ “easy‐to‐integrate” structure,^14^ and high compatibility with various types of substrates, even temperature‐sensitive ones like plastic.^15^ Therefore, OFET memory devices could be a strong candidate for hosting a variety of next‐generation memory applications, such as flexible imaging circuits^16^ and sensor arrays.^17^

Considerable efforts have been devoted to improving the properties of OFET memory devices, such as the memory window, ON/OFF current ratio, programming/erasing voltage, switching speed, retention time, endurance capability, etc. Up to now, the performance of three main types of OFET memory devices, namely floating‐gate OFET memories,^18^, ^19^ ferroelectric OFET memories,^20^, ^21^ and polymer electret OFET memories^22^, ^23^ has depended strongly on the types of gate dielectric layer, which has made research attention predominantly focus on developing functional gate dielectrics.^24^, ^25^ However, compared to the charge storage layer, the effect of the organic semiconductor layer on memory performance has received far less attention. One structure that allows for extra tuning of memory functionality is that of heterostructure OFETs utilizing both p‐type and n‐type materials as the transistor active layers, which offers both holes and electrons in the conductive channels and thus facilitates charge trapping and de‐trapping processes, resulting in a larger memory window and lower programming/erasing voltage.^26^, ^27^, ^28^ However, organic semiconductor layers in these heterostructure OFETs only function as charge transport layers, and charge‐trapping property in the organic semiconductor layer has rarely been studied. Thus, it is significant to realize both charge transport and charge trapping in organic semiconductor layer to effectively improve memory performance and take better advantage of organic semiconductor materials in OFET memory.

In this communication, we propose a novel device concept based on quantum well‐like organic semiconductor heterostructures composed of pentacene/*N*,*N*′‐ditridecylperylene‐3,4,9,10‐tetracarboxylic diimide (P13)/pentacene that has exhibited excellent nonvolatile memory properties with a pronounced memory window of ≈60 V, high ON/OFF current ratio (*I*_ON_/*I*_OFF_) of ≈10^4^, stable data endurance characteristics of 3000 cycles and a long data retention time over 10^4^ s. The calculated retention times can extend over ten years. The thickness of the bottom pentacene layer was tuned to explore the memory mechanism of the organic heterostructures transistor memory devices (OHTMs). The electrical characterization and film morphology revealed that the memory effect is a result of charge trapping in both the discontinuous P13 embedded in the pentacene layers and the polymer electret layer. OHTMs also showed reliable four‐level data storage characteristics with the retention time of each level more than 10^4^ s. Furthermore, the trilayer organic heterostructures were successfully fabricated on flexible substrates. The flexible OHTMs exhibited excellent mechanical properties and showed good memory performance even after 10 000 bending cycles.

OHTMs were fabricated in bottom‐gate and top‐contact configuration, as illustrated in **Figure** **1**a. These memory devices were prepared on heavily doped n‐type Si wafer with 300 nm thick SiO_2_ layer which served as the gate electrode and gate insulator layer, respectively. A thin layer of poly(4‐vinylphenol) (PVP) was deposited via spin‐cast followed by thermal treatment at 80 °C for 1 h. In this study, the molecules employed are pentacene for the p‐type and P13 for the n‐type organic semiconductor layer, respectively. These two materials were selected because both are well‐studied charge transport materials and are widely used in ambipolar OFETs.^29^, ^30^, ^31^, ^32^ The molecular structures and the relative positions of the highest occupied and lowest unoccupied molecular orbitals (HOMO and LUMO, respectively) energy levels of pentacene and P13 are shown in Figure 1b. Layered structures were grown by sequentially depositing 30 nm thick pentacene, 10 nm thick P13, and 18 nm thick pentacene, forming a quantum well‐like structure due to the lower LUMO energy level of P13 than that of pentacene. Finally, 50 nm thick source and drain electrodes were deposited by thermal evaporation of Au through the metal shadow mask. For further details, please see the Experimental Section. A representative set of output and transfer characteristics of the OHTMs is shown in Figures S1 in the Supporting Information. The devices showed hole‐transport‐dominated ambipolar characteristics with a hole mobility (*µ*_h_), threshold voltage (*V*_TH_), and current ON/OFF ratio of 0.23 cm^2^ V^−1^ s^−1^, −3.21 V, and 10^4^, respectively. Due to the active layer and the interface between the organic semiconductor layers being crucial to charge transport in OFETs,^33^ the film morphologies of a 30 nm thick bottom pentacene layer, 10 nm thick P13 layer and 18 nm thick top pentacene layer characterized by atomic force microscopy (AFM) are shown in Figure 1c. The bottom pentacene film deposited on PVP exhibits large grain sizes with a high degree of crystallinity, contributing to good hole transport behavior. In contrast, the morphology of the 10 nm thick P13 film grown on the bottom pentacene layer shows a discontinuous island‐like film, which is responsible for poor electron transport. The shifts in the transfer curve of OHTMs were clearly observed after applying appropriate gate voltages (*V*_G_) (see **Figure** **2**a). Memory window (Δ*V*_TH_) is defined as the difference between the *V*_TH_ of the programmed and erased states, which is 63.5 V for the OHTMs. We can calculate the charge trapping density (Δ*n*) using the equation Δ*n* = Δ*V*_TH_·*C*_i_/*e*, where *C*_i_ is the capacitance per unit area of the dielectric layer and *e* is the elementary charge. The charge trapping density in this memory device is estimated to be about 4.35 × 10^12^ cm^−2^. This result suggests that our approach is a promising strategy for realizing multilevel characteristics owing to the high charge storage capacity.

**Figure 1:**
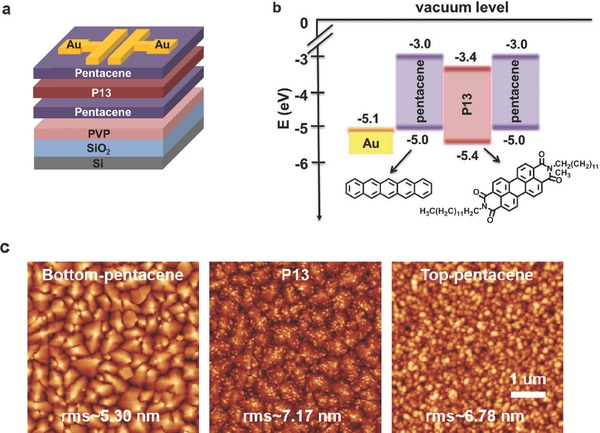
a) Schematic illustration of the OHTM. b) Energy level diagram for pentacene/P13/pentacene heterostructures. c) 5 µm × 5 µm AFM topographies for 30 nm thick bottom pentacene layer onto PVP layer, 10 nm thick P13 layer onto bottom pentacene layer and 18 nm thick top pentacene layer onto P13 layer, respectively.

**Figure 2:**
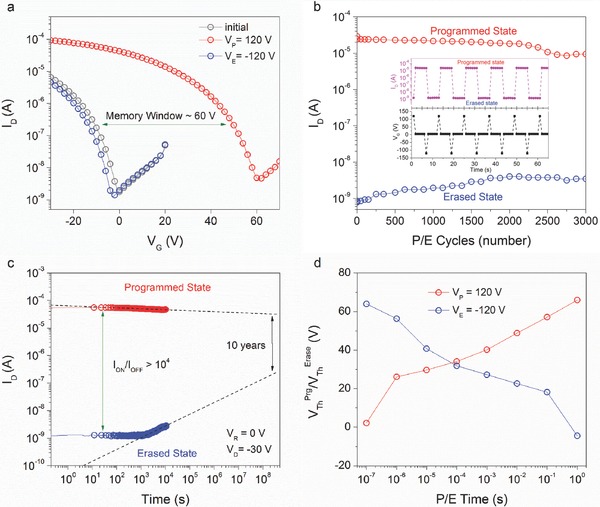
Nonvolatile memory characteristics of the OHTMs. a) Transfer curves of the OHTMs for the programming and erasing processes. b) Endurance characteristics of the OHTMs. (inset) Reversible switching behavior of the memory device in a series of programming (*V*_G_ = 120 V for 1 s), reading (*V*_G_ = 0 V, *V*_D_ = −30 V for 4 s), and erasing (*V*_G_ = −120 V for 1 s) processes. c) Retention characteristics of the OHTMs. d) Threshold voltage shift as a function of the duration of the programing and erasing pulse time of the OHTMs.

Since the stability and reliability are of particular concern in nonvolatile OFET memory devices, the programming/erasing endurance and retention capabilities of OHTMs were investigated. The inset of Figure 2b shows a set of representative data of the endurance characteristics measured from the OHTMs. The OHTMs exhibited good endurance over 3000 cycles (see Figure 2b), indicating a high reliability and stability. The retention characteristics of the OHTMs were obtained by measuring the drain current (*I*_D_) values in the programmed and erased states separately, as shown in Figure 2c. Both programmed and erased states can be well maintained for more than 10^4^ s with a high *I*_ON_/*I*_OFF_ of more than 10^4^. Further extending the fitting curve, the OHTMs still have an *I*_ON_/*I*_OFF_ of ≈10 ^2^ after ten years. It is worth noting that *V*_G_ = 0 V was applied as the reading voltage (*V*_R_), which is the optimum read voltage for memory devices with the smallest external effects and lowest power consumption. Figure 2d shows the threshold voltage shift as a function of the duration of the programming and erasing pulses for the OHTMs. The values of *V*_th,PROGRAM_ and *V*_th,ERASE_ increased with longer programming/erasing pulse durations. We summarized the key characteristics of OFET memory in this work compared with some reported works in Table S1 in the Supporting Information. It clearly demonstrates that the OHTMs are promising candidates for high‐performance nonvolatile memory in terms of charge storage capacity, reliability, and stability. The high operating voltage caused by the 300 nm thick SiO_2_ dielectric layer can be reduced by using high‐k dielectric materials or thinner dielectric layers, such as HfO_2_, AlO*_x_*, and self‐assembled monolayers (SAMs).^17^, ^19^, ^34^, ^35^

To investigate the effect of organic trilayer heterostructures on the memory performance, different OFETs were fabricated as control devices: (i) OFET based on a single pentacene layer, (ii) bilayer OFET with active layers in either bottom‐P13/top‐pentacene or bottom‐pentacene/top‐P13 structure, and (iii) trilayer OFET on bare SiO_2_ substrate, respectively (Figure S2, Supporting Information). The device based on a single pentacene layer showed negligible shift in transfer curve after a programming voltage of 120 V was applied (Figure S2a). The bilayer OFET with active layers in either bottom‐P13/top‐pentacene (Figure S2b) or bottom‐pentacene/top‐P13 (Figure S2d) structure showed large and reversible shifts of *V*_TH_ under the same bias conditions. This indicates that the organic heterostructures composed of P13 and pentacene allow to program and erase the memory device more effectively compared to the OFET based on a single pentacene layer.^36^ The memory effect is considered to originate from the electron trapping in the PVP layer. However, the programmed‐state current of the bottom‐P13/top‐pentacene device, and the erased‐state current of the bottom‐pentacene/top‐P13 device degraded rapidly, as shown in Figure S2c and S2e. The decreasing programmed‐state current of the bottom‐P13/top‐pentacene device is likely to be attributed to easily released electrons in the continuous P13 layer grown on PVP and the direct contact between the P13 and PVP layers. The obvious current increase in the erased state of the bottom‐pentacene/top‐P13 device originates from the bias effect of the high *V*_R_. Additionally, we fabricated trilayer OFET on bare SiO_2_ substrate to further demonstrate the role of P13 in charge storage. As shown in Figure S2f, the device showed a large positive *V*_TH_ of 28.5 V in its initial state and a high leakage current over 10^−8^ A due to a large density of defect states at the SiO_2_ surface. Moreover, the device exhibited a memory window of 14.5 V and the corresponding Δ*n* of 1.04 × 10^12^ cm^−2^. From the results of the control experiments, we conclude that the PVP layer plays a major role in charge trapping, and the discontinuous P13 not only provides electrons for realizing reversible programming/erasing process but also contributes to the high charge storage capacity of the memory device.

In order to further investigate the memory mechanism, we studied the memory behaviors of OHTMs with different bottom pentacene layer thicknesses (3, 12, 22, 30, and 50 nm). The shifts in transfer curves of these OHTMs under the same bias conditions are shown in **Figure** **3**a–e, and their electrical parameters are summarized in Table S2. For increasing bottom pentacene layer thickness from 3 to 50 nm, the hole transport of the OHTMs is enhanced, whereas electron transport is reduced, eventually resulting in hole‐transport‐dominated characteristics, which contributes to a higher ON/OFF current ratio between the programmed and erased states. It is worth noting that, as the thickness of the bottom pentacene layer increases, the memory window also increases, from 18.5 V for the OHTM with a 3 nm thick layer to 63.5 V for the OHTM with 30 nm thick layer, until the thickness of the bottom pentacene layer reaches 50 nm. Additionally, *I*_ON_/*I*_OFF_ reaches its highest value of 2.4 × 10^4^ when read at 0 V for the OHTM with a 30 nm thick layer. As shown in Figure 3f, when the pentacene thickness is less than 12 nm, faceted terrace‐like layered structures can be observed. Unlike the planar growth of the thin pentacene layer, the pentacene exhibits island‐type growth when the film is thicker than 12 nm. As revealed in the AFM measurements, the bottom pentacene film shows larger grain sizes and a higher degree of crystallinity as the thickness increases, which is consistent with the increase in hole mobility. However, this also results in larger grain boundaries and higher surface roughness. The P13 film is strongly affected by the morphology of the bottom pentacene layer, as P13 has a much smaller grain size than pentacene and would be deposited into the pentacene grain boundaries.^37^ P13 has a relatively low‐lying LUMO energy level compared to that of pentacene, thus pentacene/P13/pentacene heterostructures form a quantum well‐like structure, providing electron trapping sites in the discontinuous P13 film. Although more electrons would be blocked from injecting into the PVP layer as the thickness of the bottom pentacene layer increases, the degree of penetration of P13 into pentacene would be larger for thicker pentacene layers with larger grain boundaries and higher surface roughness. This may lead to an increased charge trapping capacity in a more discrete P13 film, further resulting in an overall increased charge storage capacity until the thickness of the bottom pentacene layer exceeds 30 nm, which is thick enough to block most electrons from transferring into the PVP layer, and thus the magnitude of increase in Δ*V*_th_ begins to decrease. Figure S3 in the Supporting Information shows the threshold voltage shifts as a function of the programming voltages for the OHTMs with different bottom pentacene layer thicknesses. A series of programming voltages from 0 to 120 V was applied to these OFET memory devices in steps of 20 V. When the thickness of the bottom pentacene layer is increased from 3 to 30 nm, the magnitude of the increase in Δ*V*_th_ gradually increased when subjected to the same programming voltage. However, as the thickness of the bottom pentacene layer was further increased to 50 nm, the magnitude of the increase in Δ*V*_TH_ gradually reduced. The results suggest that the shift in *V*_TH_ originates from the combined effects of the charge trapping in the discontinuous P13 and the PVP layer.

**Figure 3:**
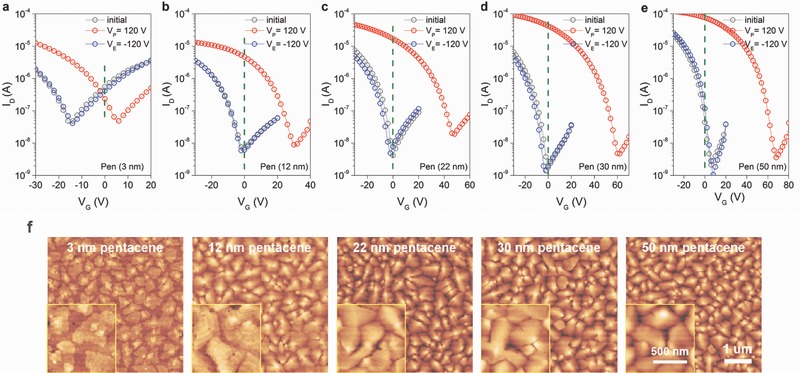
Transfer curves of the OHTMs with a) 3, b) 12, c) 22, d) 30, and e) 50 nm thick bottom pentacene layer for the programming and erasing processes. f) AFM images of bottom pentacene layer of different thicknesses. Root mean squared (*R*_RMS_) values of the roughness as calculated from the AFM images were 1.14 nm for 3 nm thick pentacene, 2.99 nm for 12 nm thick pentacene, 4.45 nm for 22 nm thick pentacene, 5.30 nm for 30 nm thick pentacene and 6.66 nm for 50 nm thick pentacene respectively, which were estimated from the surface area of 5 µm × 5 µm.

Although the exact charge storage process is not fully understood, here we propose a possible mechanism. As a positive voltage is applied to the OHMTs, electrons are generated at the P13/pentacene interface, and most of the electrons are transferred into the PVP layer. Meanwhile, a few electrons are confined in the discontinuous P13 owing to the quantum well‐like heterostructures composed of pentacene/P13/pentacene. The trapped electrons in both P13 and PVP are preserved, inducing an increased density of holes in the pentacene layer. As a result, the transfer curve of OHTMs shifts to the positive direction. In this case, the memory devices present a high conductance state. When the OHTMs are subjected to a negative voltage, the trapped electrons are detrapped and combine with holes from the active layer. Then, the transfer curve of OHTMs shifts to the negative direction, and the devices exhibit a low conductance state.

Multilevel data storage has attracted growing attention because it can further increase memory capacity per unit area without reducing the cell size, which could overcome the scale limitations of lithography technology. As reported in previous studies, the energy levels of trapped sites in polymer electret presumably present Gaussian distribution.^38^, ^39^ Therefore, the number of trapped charges in the charge trapping sites in the PVP layer can be modulated by applying different external gate voltages. Due to the large memory window, high ON/OFF current ratio, and highly stable retention capability of our memory device, we are able to achieve multilevel memory characteristics with distinct current levels. **Figure** **4**a shows the *I*_D_ levels of an OHTM at different gate voltages. Four distinguishable *I*_D_ levels can be clearly observed, including one OFF state (−120 V) and three ON states (60, 80, and 120 V), which can be read separately in a single transistor. Furthermore, the corresponding retention characteristics of the four electrical conductance states were measured, as shown in Figure 4b. The OHTMs exhibited distinct four‐level electrical conductance states with highly reliable retention time of about 10^4^ s, and the *I*_ON_/*I*_OFF_ between any two of the electrical conductance states was more than 10^1^. We also achieved reversible transfer curve shifts and multilevel data storage of OHTMs with other modified layers including polystyrene (PS), poly(methyl methacrylate) (PMMA), and octadecyltrichlorosilane (OTS) SAMs. The multilevel retention characteristics of OHTMs with different modified layers are shown in Figure S4 in the Supporting Information. All OHTMs had reliable multilevel electrical conductance states, and some even showed five electrical conductance states. Flexible OHTMs were also fabricated using PMMA as the gate insulator layer and aluminum (Al) as the gate electrode on flexible poly(ethylene terephthalate) (PET) substrates, as shown in the inset of Figure 4c. Figure 4c shows the programming and erasing characteristics of the flexible OHTMs. With the application of programming voltage of 100 V and an erasing voltage of −140 V for 1 s respectively, the flexible OHTMs show reversible shifts in *V*_TH_ with Δ*V*_TH_ and *I*_ON_/*I*_OFF_ of over 30 V and 10^2^, respectively. Figure 4d shows the variation of *V*_TH_ in the programming/erasing state as a function of the mechanical bending cycles with a bending radius of 10 mm. The variation in *V*_TH_ in the programming/erasing state of the flexible OHTMs is stable even after 10^4^ bending cycles. A corresponding photograph of flexible OHTMs under mechanical bending is presented in the inset of Figure 4d. The bending test results show that the flexible heterostructure OFET memories could still operate well after successive bending cycles. Therefore, the flexible OHTMs could be considered as promising candidates for future flexible memory devices.

**Figure 4:**
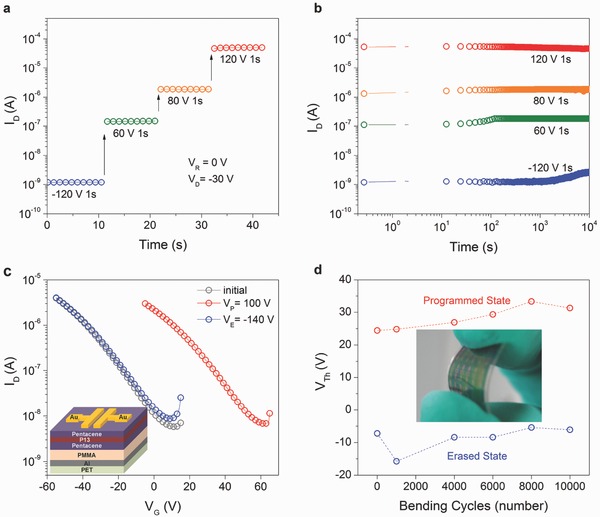
a) Corresponding drain current levels and b) Retention characteristics of the OHTMs at different programming voltages (*V*_G_ = 60, 80, 120, −120 V) which is read at *V*_G_ = 0 V, *V*_D_ = −30 V. c) Programming and erasing characteristics of the flexible OHTMs. (inset) Schematic illustration of the flexible OHTMs based on PET substrate. d) Variation in *V*_TH_ at the programming/erasing state as a function of mechanical bending cycles with a bending radius of 10 mm. (inset) A photograph of a flexible OHTM under mechanical bending.

In conclusion, we have realized the unique cooperation of charge transport and charge trapping processes occurring in the quantum well‐like pentacene/P13/pentacene organic heterostructure semiconductor layers. The synergistic effects of charge trapping in the discontinuous n‐type P13 embedded in the pentacene layer and the charge‐trapping property of the PVP layer remarkably improve the memory performance. In addition, the trilayer organic heterostructures have also been successfully applied to multilevel and flexible nonvolatile memory devices, showing that OHTMs feature high‐density storage and mechanical flexibility. Our results provide a novel design strategy to achieve high‐performance nonvolatile OFET memory devices and allow potential applications for different combinations of various organic semiconductor materials in OFET memory.

## Experimental Section

Pentacene, P13, PVP (weight‐average molecular weight Mw = 11 000), PMMA (weight‐average molecular weight Mw = 350 000), PS (weight‐average molecular weight Mw = 250 000), and OTS were purchased from Sigma‐Aldrich and used without further purification. All OFETs were fabricated in bottom‐gate and top‐contact configuration. For rigid devices, heavily doped n‐type Si wafer with 300 nm thick SiO_2_ served as gate electrode and gate insulator layer, respectively. The substrates were cleaned sequentially in an ultrasonic bath with acetone, isopropanol, and deionized water for 5 min each and dried at 100 °C for 10 min. For OHTMs based on polymer electret layer, PVP (or PS, PMMA) layer was prepared by spin‐coating from the solution of the polymer in toluene (3 mg mL^−1^) on the cleaned SiO_2_ substrates at a spin‐speed of 3000 rpm for 1 min; for OHTMs modified with SAM‐OTS, SiO_2_ substrates were immersed in OTS solution with toluene as solvent (2 mg mL^−1^) for 12 h, and then rinsed with toluene. Subsequently, the substrates were transferred in the oven to bake for 1 h at 80 °C in the air. After that, active layers (50 nm thick pentacene for the OFET based on a single pentacene layer, 10 nm thick P13, and 18 nm thick pentacene for the bilayer OFET based on bottom‐P13/top‐pentacene, 30 nm thick pentacene, and 10 nm thick P13 for the bilayer OFET based on bottom‐pentacene/top‐P13, or 30 nm thick pentacene, 10 nm thick P13 and 18 nm thick pentacene for the OHTMs) were thermally evaporated sequentially at a pressure of ≈5 × 10^−4^ Pa onto the substrates at a deposition rate of 1 Å s^−1^. The devices were completed by the formation of Au source and drain electrodes through the metal shadow mask with the channel length (*L*) and channel width (*W*) of 100 and 2000 µm, respectively. For flexible OHTMs, 70 µm thick PET sheet was used as substrate, which was cleaned sequentially with acetone, ethanol, and deionized water, and then dried at 120 °C for 20 min to improve its flexibility and thermal stability. Then, 170 nm thick Al gate electrode was thermally evaporated on the PET substrate. PMMA dielectric layer was prepared by spin‐coating from the solution of PMMA in toluene (50 mg mL^−1^) on the Al/PET substrate at 3000 rpm for 1 min, and followed by thermal treatment at 80 °C for 2 h. The residual device fabrication process was identical with the rigid OHTMs. The electrical characteristics of the devices were measured using an Agilent B1500A semiconductor parameter analyzer. All the measurements were carried out in the dark under ambient conditions. Film thickness was measured by Bruker Dektak XT stylus profiler. AFM characterizations were carried out with Bruker Scan Asyst.

## Conflict of Interest

The authors declare no conflict of interest.

## Supporting information

Supplementary
