# Supplementary material for: High‐Performance Nonvolatile Organic Field‐Effect Transistor Memory Based on Organic Semiconductor Heterostructures of Pentacene/P13/Pentacene as Both Charge Transport and Trapping Layers
Source: Adv Sci (Weinh). 2017 Jun 4;4(8):1700007. doi: 10.1002/advs.201700007 (PMC5566232; doi:10.1002/advs.201700007)
Supplement: Supplementary file 1 — Supplementary [file ADVS-4-na-s001.pdf]

Copyright WILEY-VCH Verlag GmbH & Co. KGaA, 69469 Weinheim, Germany, 2017.

ADVANCED SCIENCE
Open Access

Supporting Information

for *Adv. Sci.,* DOI: 10.1002/advs.201700007

High-Performance Nonvolatile Organic Field-Effect Transistor Memory Based on Organic Semiconductor Heterostructures of Pentacene/P13/Pentacene as Both Charge Transport and Trapping Layers

Wen Li, Fengning Guo, Haifeng Ling, Peng Zhang, Mingdong Yi, Laiyuan Wang, Dequn Wu, Linghai Xie,* and Wei Huang**

Copyright WILEY-VCH Verlag GmbH & Co. KGaA, 69469 Weinheim, Germany, 2013.

Supporting Information

Organic Heterostructures as Both Charge Transport and Trapping Layers in Organic Field-Effect Transistors for High-Performance Nonvolatile Memory

Wen Li, Fengning Guo, Haifeng Ling, Peng Zhang, Mingdong Yi, Laiyuan Wang, Dequn Wu, Linghai Xie*, Wei Huang**

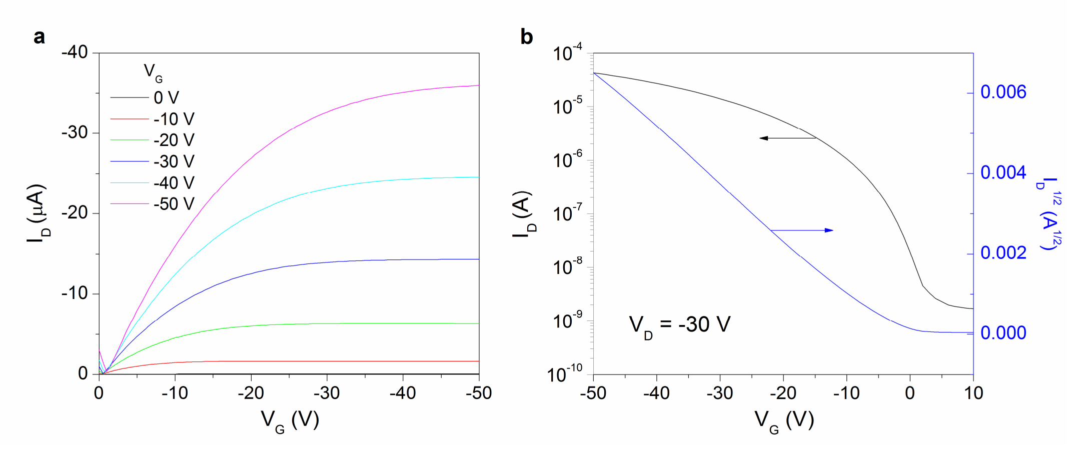

Figure S1. a) Output and b) transfer characteristics of the OHTM.

Table S1. Summary of key characteristics of OFET memory in this paper compared with those reported in the literature.

Substrate /Dielectric(s) Type	Organic Semiconductor Material(s)	Proposed Mechanism Type a)	Charge Storage Elements	Mobility [cm^2/Vs]	V_P/V_E [V]	t_P/t_E [s]	Memory Window [V]	Charge Trapping Density [$x10^{12}$]	Memory Ratio	P/R/E Cycles c)	Retention Time	REF
Glass/PVA	PCBM	PE	PVA	$9x10^{-2}$ (electron)	±50	~1	14	0.26*	10^2	200 s	15 h	[S1]
Si/300 nm SiO_2	pentacene	PE	PaMS	0.51 (hole)	+200/ -100	10^{-6}	90	5	10^5	-	$3x10^4$ s	[S2]
PI/PVP	tips-pentacene	FE	PVDF	0.65 (hole)	±15	sweep	~20	-	10^4	-	10^5 s	[S3]
PEN/AlO_x+SAM	pentacene	FG	Al film	-	-6/+3	1	2.5	4.9*	10^3	10^4	10^4 s	[S4]
PES/cross-linked PVP	pentacene	FG	Au NPs	0.25 (hole)	±90	1	10	0.56*	>10^2	700	10^5 s (>1 y)	[S5]
Glass/Al_2O_3	a,a´-DH6T	FG	SAM	0.04 (hole)	±2	30	~0.1	0.49*	2.4	-	6 h	[S6]
PDMS coated banknote	pentacene	FE	P(VDF-TrFE)	0.12 (hole)	±15	sweep	8	-	$5x10^3$	~20 s	10^4 s	[S7]
Si/300 nm SiO_2	pentacene/C_{60}	FG	Au NPs	$2.5×10^{-3}$ (hole) $1.2×10^{-2}$ (electron)	±40	4	~10	0.72	-	10^3	10^4 s	[S8]
Glass/P(VDF-TrFE)	PC12TV12T	PE	PVN	0.61 (hole)	±90	sweep	~92	9.07*	10^5	100 s	$3x10^3$ s (>10^7 s)	[S9]
Si/30 nm SiO_2	pen/PDI-8	PE	PS	0.2 (hole) 0.32 (electron)	±12	10^{-3}	~ 5	2.25*	~10^2	600	-	[S10]
Si/300 nm SiO_2	pentacene	FG	Ferritin NPs	0.013 (hole)	±100	10^{-5}	>20	4.02*	10^4	200	10^4 s (>10^7 s)	[S11]
PET/Al_2O_3	pentacene	FG	rGO sheets +Au NPs	0.1 (hole)	±5	1	1.95	1.89*	~10^3	10^3	10^5 s	[S12]
Si/AlO_x+SAM	pentacene	FG	GO	0.11 (hole)	+5/ -5.5	5	~0.85	2.9	~10^2	20 s	10^4 s (>1 y)	[S13]
PEN/P(VDF-TrFE)	P3HT	PE+FG	PVN+Cu NPs	$5.2×10^{-2}$ (hole)	±50	1	~43	4.8	10^4	120 s	$3x10^4$ s (>1 y)	[S14]

Si/100 nm SiO_2	pentacene	FG	Au NPs+ Pt NPs	0.53 (hole)	±45	1	18.7	~1	>10^5	600	10^4 s	[S15]
Glass/HfO_2	pentacene	FG	CuPc NPs+ N-C_{60}	1.25×10^{-2} (hole)	±5	sweep	4.2	1.8 (hole) 1.6 (electron)	8.3x10^3	500	10^4 s	[S16]
Si/300 nm SiO_2	P13	PE	polymide	0.31 (electron)	±90	1	15.5	0.87*	10^3	-	5x10^3 s	[S17]
Glass/[P(VDF-TrFE-CTFE)]	pentacene	FE	[P(VDF-TrFE-CTFE)]	0.8 (hole)	±15	sweep	19.2	-	10^3	100	3x10^3 s	[S18]
Si/300 nm SiO_2	pentacene	PE	β-phase PFO	0.13 (hole)	-80/L [b)]	1	57	3.3	10^3	300 s	10^4 s	[S19]
Si/300 nm SiO_2	pentacene	FG	Au NPs/porous PMMA	0.49 (hole)	L+80/ -150	3	43	2.87	10^5	300 s	10^4 s	[S20]
Si/PMMA	P(NDI2OD-T2)	FG	PS:P3HT	8.2×10^{-2} (hole)	±50	1	9.6	0.6*	142	500	5x10^3 s	[S21]
Glass/[P(VDF-TrFE-CTFE)]	F_{16}CuPc/pen	PE	PVP	0.35 (hole) 1.3×10^{-2} (electron)	±25	1	8.5	-	10^4	3x10^3	10^4 (>10 y)	[S22]
PEN/ P(VDF-TrFE)/PVT	DNTT	FE	P(VDF-TrFE)	7×10-2 (hole)	±200	sweep	~190*	-	10^3	100	5x10^3	[S23]
**Si/300 nm SiO_2**	pentacene **/P13/** pentacene	**PE**	**P13/PVP**	**0.23**	**±120**	**1 s**	**63.5**	**4.36**	**10^4**	**3x10^3**	**10^4 (>10 y)**	**this work**

[a)] PE: Charge trapping polymer electret, FG: Floating-gate, FE: Ferroelectric; [b)] L: Light illumination (5 mW/cm^{-2}, 410-800 nm); * Data derived from the origin data in the paper.

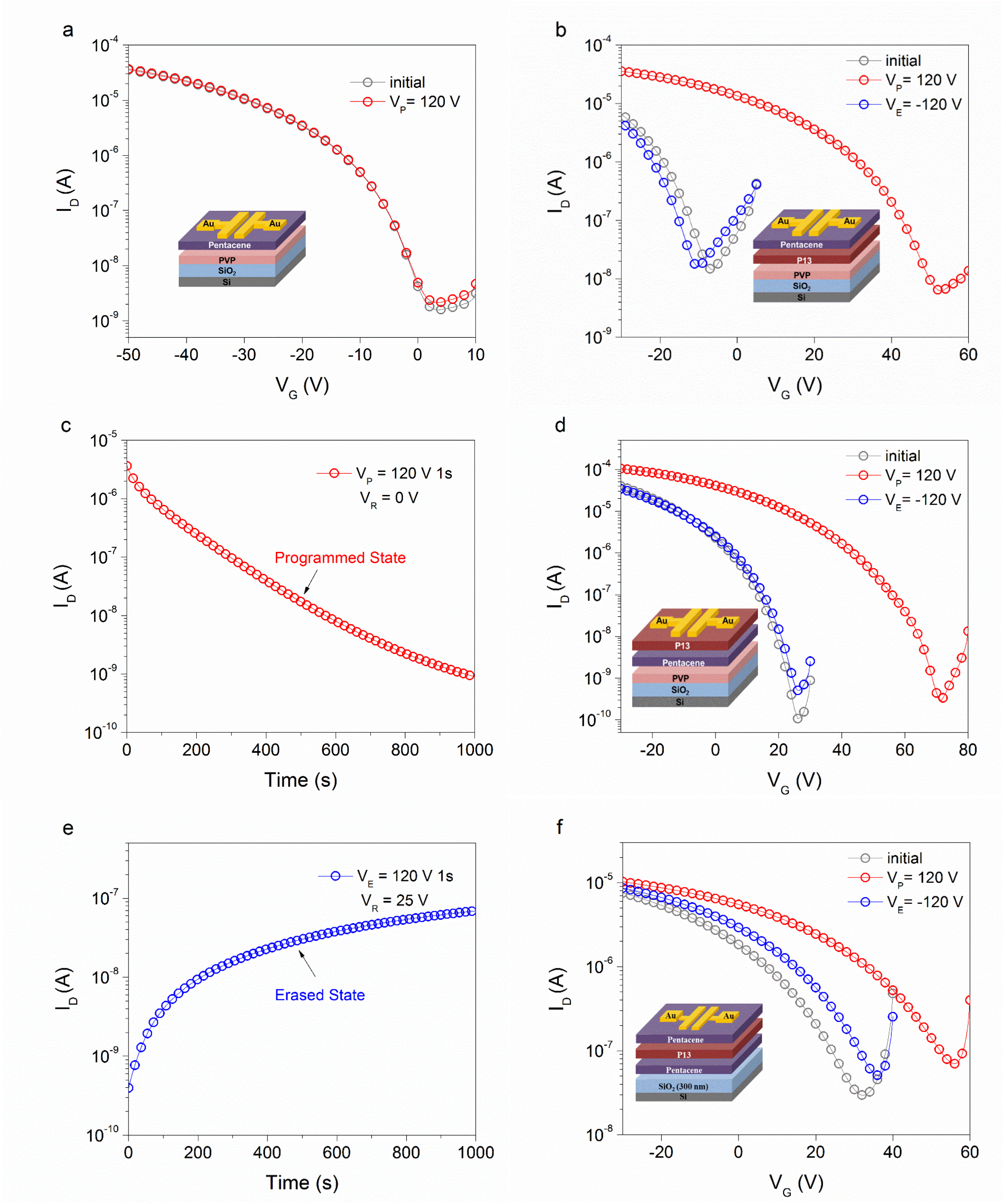

Figure S2. a) Transfer curves of the OFET based on a single pentacene in the initial state and programmed state. (inset) Schematic illustration of the OFET based on a single pentacene layer. b) Transfer curves of the bilayer OFET based on bottom-P13/top-pentacene for the programming/erasing processes. (inset) Schematic illustration of the bilayer OFET based on bottom-P13/top-pentacene. c) Retention characteristics of the bilayer OFET based on bottom-P13/top-pentacene in the programmed state. d) Transfer curves of the bilayer OFET based on bottom-pentacene/top-P13 for the programming/erasing processes. (inset) Schematic illustration of the bilayer OFET based on bottom-pentacene/top-P13. e) Retention characteristics of the bilayer OFET based on bottom-pentacene/top-P13 in the erased state. f) Transfer curves of the trilayer OFET fabricated on bare SiO_2. (inset) Schematic illustration of the trilayer OFET fabricated on bare SiO_2.

Table S2. μ_h, μ_e, V_{TH}, ΔV_{TH} values, Δn and I_{ON}/I_{OFF} of OHTMs with different bottom pentacene thicknesses.

Sample	μ_h [b)] [cm^2 V^{-1} s^{-1}]	μ_e [c)] [cm^2 V^{-1} s^{-1}]	V_{TH} [d)] [V]	ΔV_{TH} [V]	Δn [$\times 10^{12}$ cm^{-2}]	I_{ON}/I_{OFF}
Pen [a)] (3 nm)/P13/Pen	0.10	0.06	-13.50	18.5	1.27	-
Pen (12 nm)/P13/Pen	0.12	0.0019	-6.46	32.7	2.25	6.97×10^{2}
Pen (22 nm)/P13/Pen	0.13	0.0011	-5.69	47.6	3.27	2.22×10^{3}
Pen (30 nm)/P13/Pen	0.23	-	-3.21	63.5	4.36	2.40×10^{4}
Pen (50 nm)/P13/Pen	0.31	-	1.61	58.2	4.00	6.52×10^{2}

[a)] Pen represents pentacene; [b)] μ_h is the hole mobility; [c)] μ_e is the electron mobility; and [d)] V_{TH} is the threshold voltage for p-type behavior that obtained at V_D = -30 V.

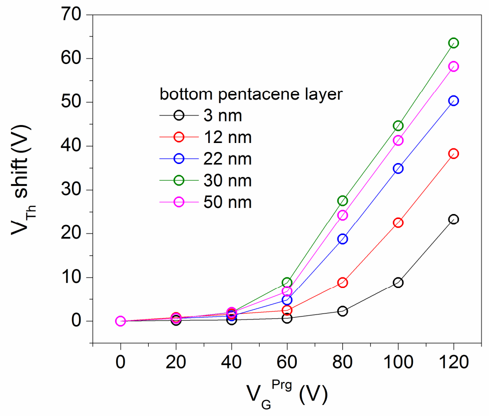

Figure S3. Threshold voltage shifts as a function of programming gate voltage for the OHTMs with different bottom pentacene thicknesses.

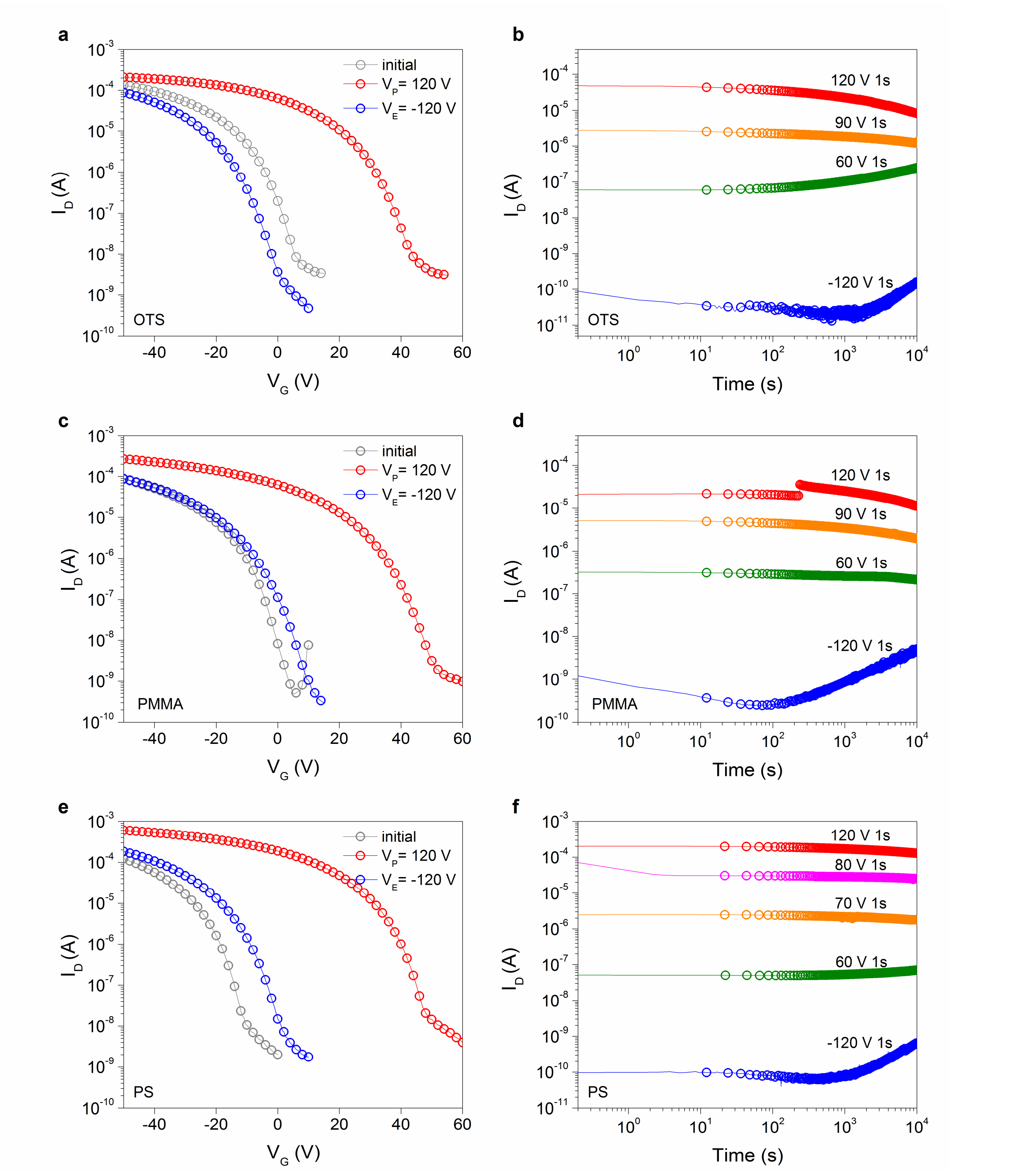

Figure S4. a) Transfer curves of the OHTM with SAM-OTS for the programming/erasing processes. b) Retention characteristics of the OHTM with SAM-OTS at different programming voltages which is read at V_G = 0 V, V_D= -30 V. c) Transfer curves of the OHTM with PMMA for the programming/erasing processes. d) Retention characteristics of the OHTM with PMMA at different programming voltages. e) Transfer curves of the OHTM with PS for the programming/erasing processes. f) Retention characteristics of the OHTM with PS at different programming voltages.

References

[S1] Th. B. Singh, N. Marjanović, G. J. Matt, N. S. Sariciftci, *Appl. Phys. Lett.* **2004**, *85*, 5409.

[S2] K.-J. Baeg, Y.-Y. Noh, J. Ghim, S.-J. Kang, H. Lee, D.-Y. Kim, *Adv. Mater.* **2006**, *18*, 3179.

[S3] S. J. Kang, Y. J. Park, I. Bae, K. J. Kim, H.-C. Kim, S. Bauer, E. L. Thomas, C. Park, *Adv. Funct. Mater.* **2009**, *19*, 2812.

[S4] T. Sekitani, T. Yokota, U. Zschieschang, H. Klauk, S. Bauer, K. Takeuchi, M. Takamiya, T. Sakurai, T. Someya, *Science* **2009**, *326*, 1516.

[S5] S.-J. Kim, J.-S. Lee, *Nano Lett.* **2010**, *10*, 2884.

[S6] M. Burkhardt, A. Jedaa, M. Novak, A. Ebel, K. Vo¨itchovsky, F. Stellacci, A. Hirsch, M. Halik, *Adv. Mater.* **2010**, *22*, 2525.

[S7] M.A. Khan, U. S. Bhansali, H. N. Alshareef, *Adv. Mater.* **2012**, *24*, 2165.

[S8] Y. Zhou, S.-T. Han, Z.-X. Xu, V. A. L. Roy, *Adv. Mater.* **2012**, *24*, 1247.

[S9] K.-J. Baeg, D. Khim, J. Kim, B.-D. Yang, M. Kang, S.-W. Jung, I.-K. You, D.-Y. Kim, Y.-Y. Noh, *Adv. Funct. Mater.* **2012**, *22*, 2915.

[S10] Y. Guo, J. Zhang, G. Yu, J. Zheng, L. Zhang, Y. Zhao, Y. Wen, Y. Liu, *Org. Electron.* **2012**, *13*, 1969.

[S11] B. J. Kim, Y. Ko, J. Ho Cho, J. Cho, *small* **2013**, *9*, 3784.

[S12] S.-T. Han, Y. Zhou, C. Wang, L. He, W. Zhang, V. A. L. Roy, *Adv. Mater.* **2013**, *25*, 872.

[S13] T.-W. Kim, N. Cernetic, Y. Gao, S. Bae, S. Lee, H. Ma, H. Chen, A. K.-Y. Jen, *Org. Electron.* **2014**, *15*, 2775.

[S14] M. Kang, D. Khim, W.-T. Park, J. Kim, J. Kim, Y.-Y. Noh, K.-J. Baeg, D.-Y. Kim, *Sci. Rep.* **2015**, *5*, 12299.

[S15] J.-Y. Zhang, L.-M. Liu, Y.-J. Su, X. Gao, C.-H. Liu, J. Liu, B. Dong, S.-D. Wang, *Org. Electron.* **2015**, *25*, 324.

[S16] H.-C. Chang, C. Lu, C.-L. Liu, W.-C. Chen, *Adv. Mater.* **2015**, *27*, 27.

[S17] Y.-F. Wang, M.-R. Tsai, Y.-S. Lin, F.-C. Wu, C.-Y. Lin, H.-L. Cheng, S.-J. Liu, F.-C. Tang, W.-Y. Chou, *Org. Electron.* **2015**, *26*, 359.

[S18] L. Xiang, W. Wang, W. Xie, *Sci. Rep.* **2016**, *6*, 36291.

[S19] H. Ling, J. Lin, M. Yi, B. Liu, W. Li, Z. Lin, L. Xie, Y. Bao, F. Guo, W. Huang, *ACS*

Appl. Mater. Interfaces **2016**, *8*, 18969.

[S20] M.Yi, J. Shu, Y. Wang, H. Ling, C. Song, W. Li, L. Xie, W. Huang, *Org. Electron.* **2016**, *33*, 95.

[S21] W. Wang, K. L. Kim, S. M. Cho, J. H. Lee, C. Park, *ACS Appl. Mater. Interfaces* **2016**, *8*, 33863.

[S22] L. Xiang, J. Ying, J. Han, L. Zhang, W. Wang, *Appl. Phys. Lett.* **2016**, *108*, 173301.

[S23] D. Thuau, M. Abbas, G. Wantz, L. Hirsch, I. Dufour, C. Ayela, *Org. Electron.* **2017**, *40*, 30.
